# Bladder Endometriosis Resolved by Robotic-Assisted Partial Cystectomy

**DOI:** 10.7759/cureus.104573

**Published:** 2026-03-02

**Authors:** Adrianna Gorniak, Marie-Claire Leaf, Andrew Gabrielson, Sunil H Patel, Anja Frost

**Affiliations:** 1 Gynecology and Obstetrics, Johns Hopkins University, Baltimore, USA; 2 Gynecology, Orange Coast Women's Medical Group, Laguna Hills, USA; 3 Urology, Johns Hopkins University, Baltimore, USA

**Keywords:** bladder endometriosis, deep infiltrating endometriosis (die), endometriosis, endometriosis excision, partial cystectomy, robotic bladder repair

## Abstract

This is a case report of a 31-year-old G1P1 with deep infiltrating endometriosis (DIE) of the bladder. She presented with bladder spasms, dysuria, and urinary frequency that worsened during her menses. On magnetic resonance imaging (MRI), she was found to have a 2.7 cm bladder dome mass and a 1.9 cm left-sided endometrioma. Biopsy of the bladder mass by urology was consistent with endometriosis. The patient failed medical management with Depo-Provera and GnRH antagonists and desired to proceed with surgical intervention. She ultimately underwent a robotic-assisted partial cystectomy for DIE of the bladder as well as ovarian cystectomy and excision of endometriosis in conjunction with gynecology and urology teams. The bladder endometriotic nodule was dissected circumferentially and the cystotomy was closed in two layers using absorbable 3-0 V-Loc. At her post-operative visit, the patient’s symptoms had significantly improved. This case demonstrates that deep infiltrating bladder endometriosis should be on the differential for patients presenting with cyclic pelvic pain and urinary symptoms. Medical management is a reasonable first step depending on imaging findings, symptoms, and fertility goals; but surgical management with partial cystectomy may be warranted.

## Introduction

Endometriosis is a systemic disease, prevalent in about 10% of female patients [1], and it can necessitate multidisciplinary care for proper management [2]. Urinary tract endometriosis (UTE) is a rare subset of endometriosis, occurring in about 1% of cases, which is often associated with deep infiltrating endometriosis (DIE) in the pelvis [1,3]. The pathophysiology of UTE involving the bladder includes ectopic endometrial tissue invasion of the detrusor muscle, which occurs through chronic inflammation and fibromuscular infiltration [1,4]. Patients often present with typical symptoms of endometriosis in addition to cyclic urinary symptoms. Similar to pelvic endometriosis, medical management is first-line treatment, but surgical management may be required for adequate symptom management. For patients requiring surgery with full-thickness bladder endometriotic implants, multidisciplinary management, involving both gynecologic and urologic surgeons, is necessary as these lesions frequently require partial cystectomy to achieve symptom resolution [3,4]. We present the case of a patient with DIE of the bladder who underwent a partial cystectomy with complete resolution of symptoms. This case was previously presented as a virtual poster presentation at the American Association of Gynecologic Laparoscopists (AAGL) Global Congress on November 16, 2024.

## Case presentation

**Figure 1:**
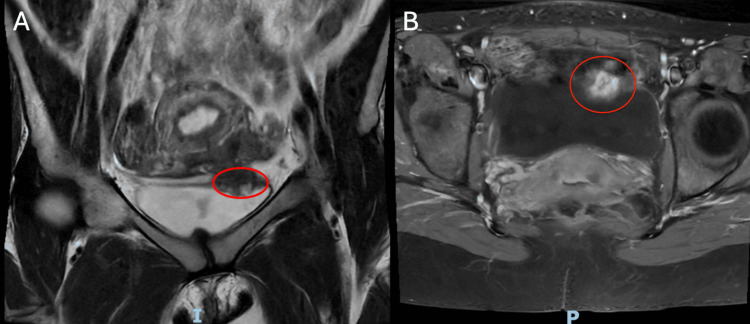
Pelvic MRI demonstrating 2.4 x 2.1 x 2.7 cm bladder dome lesion. (A) Coronal T2-weighted MRI demonstrating a hypointense lesion at the left bladder dome (red circle). (B) Axial T1-weighted MRI demonstrating multiple foci of hyperintensity (hemorrhagic content) within the lesion (red circle).

A 31-year-old G1P1 female patient was referred to the minimally invasive gynecologic surgery (MIGS) clinic for DIE of the bladder and a left-sided endometrioma. The patient’s initial symptoms prior to diagnosis and management included cyclical pelvic and urinary symptoms such as bladder spasms, dysuria, and urinary frequency despite multiple negative cultures. She had been experiencing these symptoms for one year prior to diagnosis. Notably, she did not have hematuria; however, this patient did have a long-standing history of heavy menstrual bleeding and dysmenorrhea. Prior to ultimately being diagnosed with DIE of the bladder, differential diagnosis included interstitial cystitis, bladder tumor, and secondary dysmenorrhea of uncertain etiology. The patient trialed medical therapy prior to referral to the MIGS clinic, including Depo-Provera and elagolix, a GnRH antagonist, with suboptimal control of symptoms and intolerable side effects, leading to self-discontinuation after a few months. She underwent pelvic magnetic resonance imaging (MRI), which showed a 2.4x2.1x2.7 cm T2 hypointense mass at the left-bladder dome with multiple foci of T1 hyperintensity (Figure 1). No other intraluminal bladder abnormalities were noted. There was also a 1.9x1.6 cm T1 hyperintense lesion on the left ovary consistent with an endometrioma, in addition to multiple punctuate lesions on the left broad ligament also likely representing endometrial implants.

**Figure 2:**
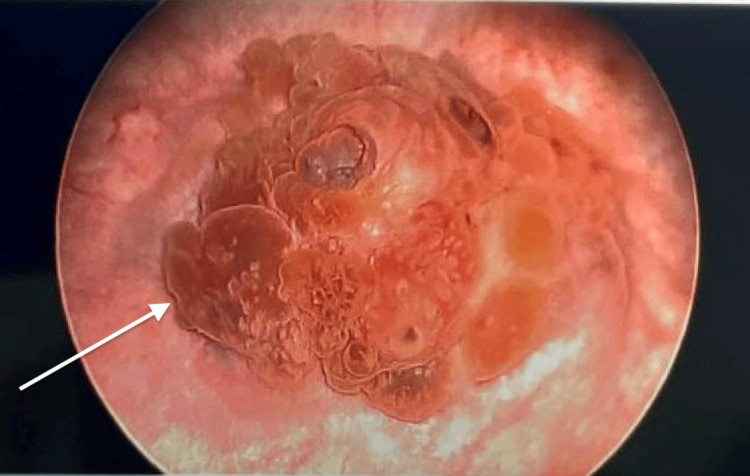
Cystoscopic view of bladder endometriosis. Cystoscopy demonstrating a 3 cm nodular lesion at the bladder dome (white arrow) consistent with endometriosis.

She then underwent cystoscopy, which demonstrated a 3 cm polypoid bladder dome mass (Figure 2) with transurethral biopsy of the bladder tumor consistent with an endometrioma. The lesion was unable to be completely resected via cystoscopy. The patient elected to undergo conservative surgical management of endometriosis for fertility preservation with both the gynecology and urology teams.

The patient underwent an uncomplicated robotic-assisted partial cystectomy, left ovarian cystectomy, and excision of endometriosis. The procedure started with incising the peritoneum and separating the bladder from its anterior attachments to the pelvis using a combination of cautery and blunt dissection. The bladder was filled via retrograde instillation of normal saline. With the bladder distended, the bladder surface was cleared off with at least a 2 cm margin to allow for tension-free closure after excision of the bladder lesion. The bladder was entered at the dome to provide adequate visualization of its entirety, and the mass was carefully excised circumferentially using robotic scissors. During dissection, the trigone was visualized to avoid any ureteral injuries. Once the mass was removed, the cystotomy was closed tension-free in two layers using absorbable 3-0 V-Loc (Medtronic, Minneapolis, MN, USA). A leak test was performed with retrograde instillation of 300 cc of saline, which confirmed a watertight closure. In addition to resection of the deep infiltrating bladder endometriosis, pelvic peritoneal endometriosis lesions were resected and an ovarian cystectomy was performed to remove a large endometrioma.

The patient was discharged on the same day of surgery with a Foley catheter in place and a short course of oxybutynin. On post-operative day seven, the patient had her Foley catheter removed and had no voiding dysfunction. On follow-up, the patient reported complete resolution of her urinary symptoms, pelvic pain, and dysmenorrhea.

## Discussion

UTE occurs in about 1% of patients with endometriosis [1]; however, of these patients, about 50% will be asymptomatic [3]. About 90% of patients will also have pelvic endometriosis implants or endometriomas [3,4]. The urinary system is the second most common site for extra-pelvic implants following the gastrointestinal system [3]. In the urinary system, the bladder is the most common site of endometriosis implants, with about 85% of UTEs implanting on the bladder, specifically the bladder dome [2-4]. In addition to the typical symptoms of endometriosis, cyclical pelvic pain and infertility, symptoms of bladder endometriosis will include primarily dysuria but also urinary frequency, recurrent urinary tract infections, hematuria, and occasionally urinary incontinence [3,4].

The diagnosis of bladder endometriosis is ultimately histologic; however, transvaginal ultrasound and/or MRI is the standard to help characterize the lesions [2-4]. On MRI, these lesions will have T2 hypointensity with occasional T1 hyperintensity signals [3,4]. If transvaginal ultrasound is performed, it is important to also image the abdomen to evaluate for further urinary endometrial implants causing hydronephrosis [3]. Cystoscopy can also be useful to rule out malignancy, confirm suspected diagnosis, and to help with surgical planning [3,4].

Patients with UTE, without hydronephrosis or ureteral obstruction, can opt for first-line treatment, which is medical management with progesterone therapy, combined estrogen progesterone contraceptives, or GnRH analogues [3,4]. For some patients with bladder endometriosis, adequate symptom management is only achieved with surgery [3]. For deeply infiltrating lesions into the bladder, the optimal surgical approach is with a minimally invasive approach and includes removal of the endometriosis lesion or partial cystectomy [4].

Following a partial cystectomy, the bladder should be closed in two layers and a Foley catheter should be left in place for a minimum of seven days [5]. A voiding cystourethrogram, low-dose CT cystogram, or three-view plain film cystogram (anteroposterior with bladder distended, anteroposterior post-drainage, and lateral post-drainage) should be considered after Foley removal if the defect is large, complex, or is being performed in a patient with prior bladder reconstruction or pelvic radiation [6,7]. If the defect is less than 2 cm and otherwise uncomplicated, a single-layer closure of the bladder defect could be permitted [5,6,8]. While traditionally smooth suture, such as Vicryl or polydioxanone (PDS), would be used to close cystotomies [8], barbed suture, such as V-Loc, used in this case, has increased tensile strength [9] and is safe to use in the repair of bladder defects [10,11]. Unless a patient has had pelvic radiation or the bladder tissue is of poor quality, a drain can typically be omitted in most cases.

## Conclusions

Bladder endometriosis should be on the differential for patients presenting with cyclic pelvic pain and urinary symptoms. Medical management is a reasonable first step; however, for patients who have failed or do not desire medical management, surgical management with partial cystectomy for deep endometrial bladder implants is reasonable. These cases should be performed with a multidisciplinary team for optimal patient outcomes given the complexity of multi-organ endometriosis.
